# Supplementary material for: Flexible genes establish widespread bacteriophage pan-genomes in cryoconite hole ecosystems
Source: Nat Commun. 2020 Sep 2;11:4403. doi: 10.1038/s41467-020-18236-8 (PMC7468147; doi:10.1038/s41467-020-18236-8)
Supplement: Supplementary file 1 — Supplementary Information [file 41467_2020_18236_MOESM1_ESM.pdf]

Flexible genes establish widespread bacteriophage pan-genomes in cryoconite hole ecosystems

Bellas et al.

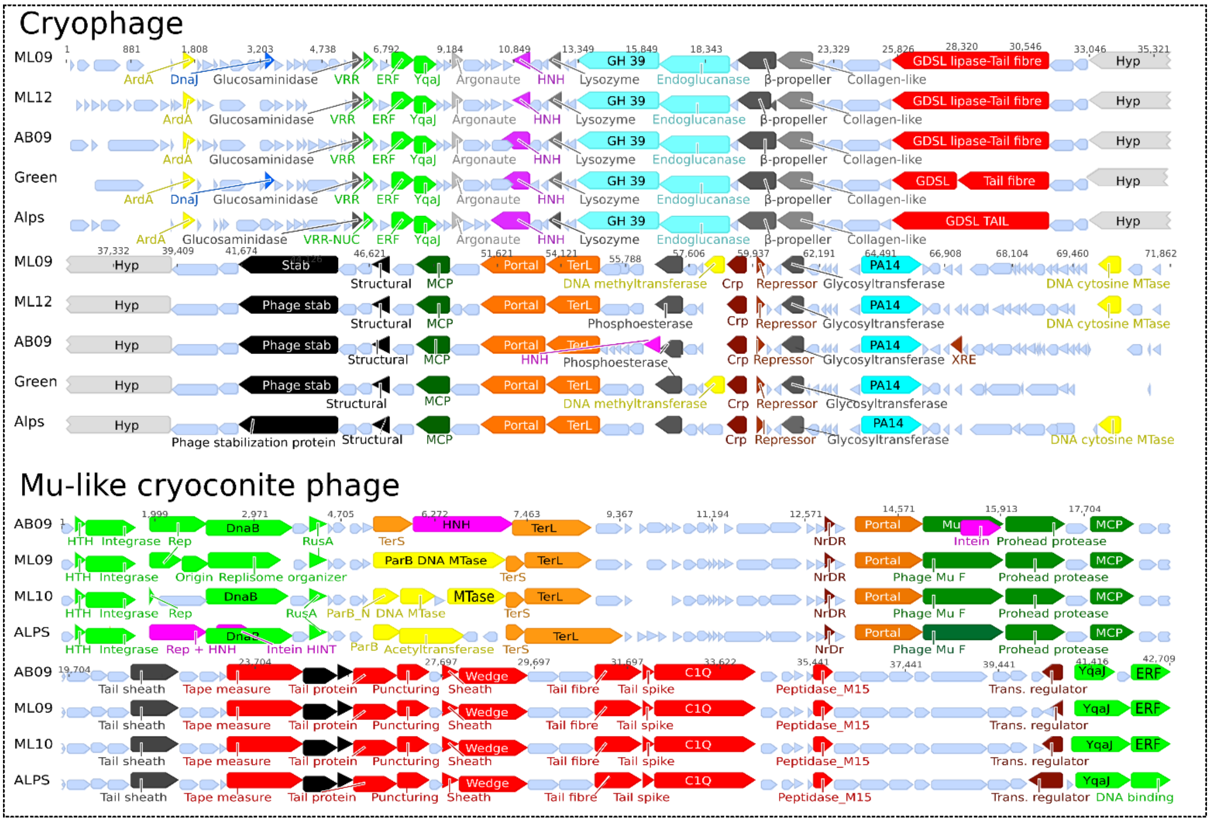

Supplementary Figure 1 Annotated alignments of Cryophage and the Mu-like phage original and reconstructed genomes. The underlying nucleotide sequences were aligned using Muscle, hence gene sizes are approximate and may include gapped regions to preserve the alignment. ArdA - Antirestriction protein, C1q – C1q domain like protein, Crp – transcriptional regulator, DnaB – DnaB helicase, ERF - Essential Recombination Function protein, GH39 - Glycoside Hydrolase 39, HNH - HNH type homing endonuclease, HYP- Hypothetical protein, MCP – Major Capsid Protein, NrDR – transcriptional repressor, PA14 – PA14 domain, ParB – Plasmid partition parB protein, Phage Mu F – capsid assembly protein F, Phage stab – Phage stabilisation protein, Portal – Phage portal protein, Rep - Replication protein, RusA – crossover junction endodeoxyribonuclease, Sheath – Baseplate sheath, TerL – Large terminase subunit, Wedge – Baseplate wedge, VRR - VRR-NUC domain, XRE – XRE family transcriptional regulator

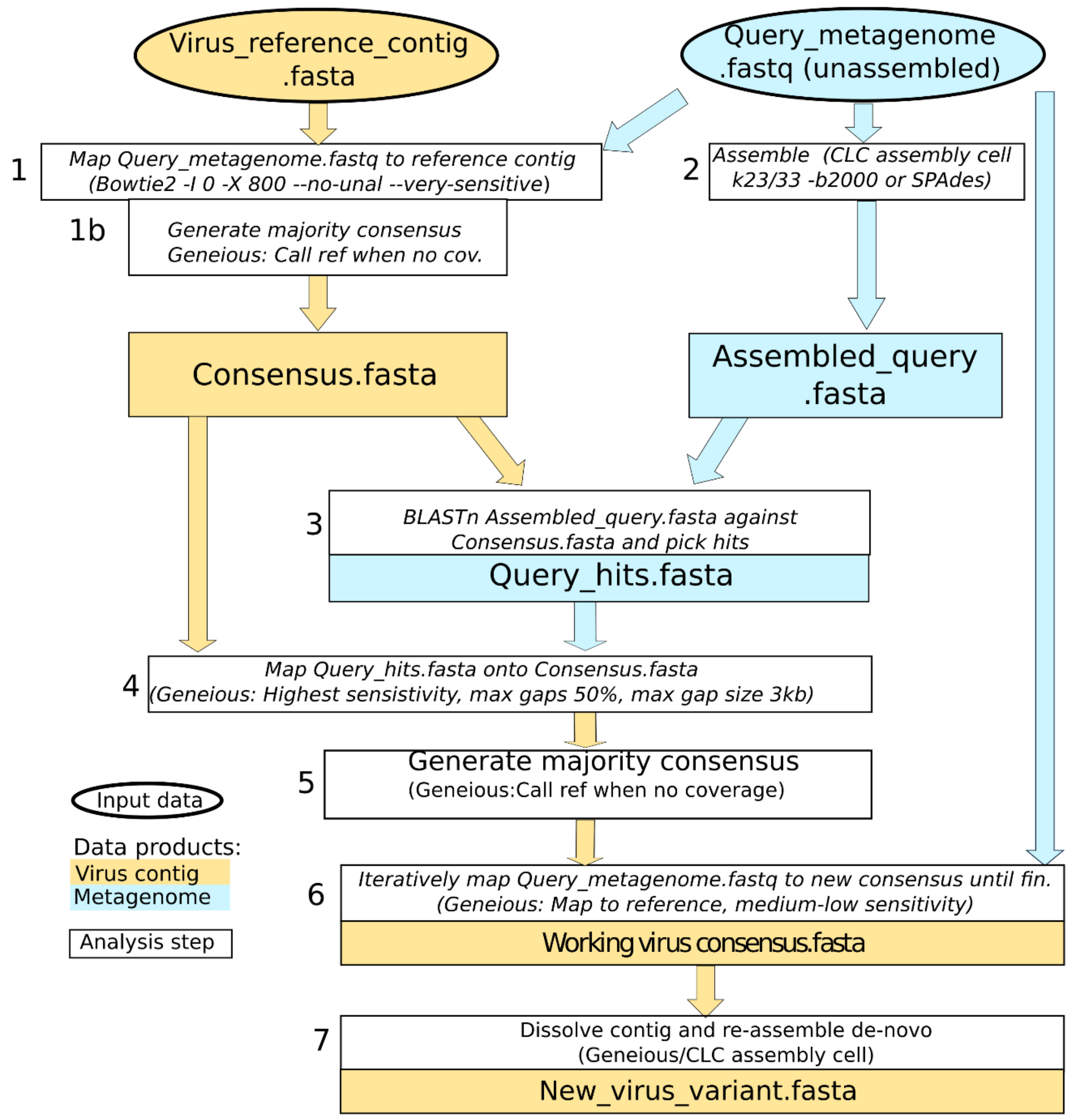

Supplementary Figure 2 Flow diagram for MGI variant reconstructions. A virus reference contig is used to recruit reads from the query metagenome (1) using Bowtie2 and a new consensus is generated (1b). The query metagenome is then mapped against the new consensus via a BLASTn search to retrieve a list of contigs (3) which are then imported into Geneious R8 and mapped against the consensus (4) to generate a new consensus (5). Query metagenomic reads were then iteratively mapped onto the working consensus (6) in Geneious until no further extension was possible. Recruited reads to the final consensus were then extracted and reassembled de novo to confirm the assembly of the new variant (7). Yellow rectangles represent to virus consensus data products; blue rectangles are the query metagenomic reads/assembled contigs which align to the reference. White rectangles and italics represents an analysis step.

Supplementary references

1. Pires, D. P., Oliveira, H., Melo, L. D. R., Sillankorva, S. & Azeredo, J. Bacteriophage-encoded depolymerases: their diversity and biotechnological applications. *Appl. Microbiol. Biotechnol.* **100**, 2141–2151 (2016).

2. Yamada, T. *et al.* A jumbo phage infecting the phytopathogen Ralstonia solanacearum defines a new lineage of the Myoviridae family. *Virology* **398**, 135–147 (2010).

3. Zhao, H. *et al.* Structure of a bacterial virus DNA-injection protein complex reveals a decameric assembly with a constricted molecular channel. *PLoS One* **11**, 1–16 (2016).

4. Garcia-Heredia, I. *et al.* Reconstructing viral genomes from the environment using fosmid clones: the case of haloviruses. *PLoS One* **7**, e33802 (2012).

5. Prokhorov, N. S. *et al.* Function of bacteriophage G7C esterase tailspike in host cell adsorption. *Mol. Microbiol.* **105**, 385–398 (2017).

6. Barbirz, S. *et al.* Crystal structure of *Escherichia coli* phage HK620 tailspike: Podoviral tailspike endoglycosidase modules are evolutionarily related. *Mol. Microbiol.* **69**, 303–316 (2008).

7. Roux, S., Krupovic, M., Poulet, A., Debroas, D. & Enault, F. Evolution and diversity of the Microviridae viral family through a collection of 81 new complete genomes assembled from virome reads. *PLoS One* **7**, e40418 (2012).

8. Rodríguez-Rubio, L., Martínez, B., Donovan, D. M., Rodríguez, A. & García, P. Bacteriophage virion-associated peptidoglycan hydrolases: Potential new enzybiotics. *Crit. Rev. Microbiol.* **39**, 427–434 (2013).

9. Chang, K. *et al.* Genomic analysis of bacteriophage ϕAB1, a ϕKMV-like virus infecting multidrug-resistant *Acinetobacter baumannii*. *Genomics* **97**, 249–255 (2011).
