# Supplementary material for: Flexible genes establish widespread bacteriophage pan-genomes in cryoconite hole ecosystems
Source: Nat Commun. 2020 Sep 2;11:4403. doi: 10.1038/s41467-020-18236-8 (PMC7468147; doi:10.1038/s41467-020-18236-8)
Supplement: Supplementary file 3 — Description of Additional Supplementary Files [file 41467_2020_18236_MOESM3_ESM.docx]

**Description of Additional Supplementary Files**

File Name: Supplementary Data 1

Description: All detected metagenomic islands in bacteriophage genomes summarised by predicted function.

File Name: Supplementary Data 2

Description: Annotation of the predicted genes from Cryophage and the Mu-like cryoconite phage.
