# Supplementary material for: Flexible genes establish widespread bacteriophage pan-genomes in cryoconite hole ecosystems
Source: Nat Commun. 2020 Sep 2;11:4403. doi: 10.1038/s41467-020-18236-8 (PMC7468147; doi:10.1038/s41467-020-18236-8)
Supplement: Supplementary file 6 — Reporting Summary [file 41467_2020_18236_MOESM6_ESM.pdf]

nature research

Corresponding author(s): Christopher Bellas
Last updated by author(s): 31/07/2020

Reporting Summary

Nature Research wishes to improve the reproducibility of the work that we publish. This form provides structure for consistency and transparency in reporting. For further information on Nature Research policies, see our Editorial Policies and the Editorial Policy Checklist.

Statistics

For all statistical analyses, confirm that the following items are present in the figure legend, table legend, main text, or Methods section.

n/a	Confirmed	
☒	☐	The exact sample size (n) for each experimental group/condition, given as a discrete number and unit of measurement
☒	☐	A statement on whether measurements were taken from distinct samples or whether the same sample was measured repeatedly
☒	☐	The statistical test(s) used AND whether they are one- or two-sided<br>*Only common tests should be described solely by name; describe more complex techniques in the Methods section.*
☒	☐	A description of all covariates tested
☒	☐	A description of any assumptions or corrections, such as tests of normality and adjustment for multiple comparisons
☐	☒	A full description of the statistical parameters including central tendency (e.g. means) or other basic estimates (e.g. regression coefficient) AND variation (e.g. standard deviation) or associated estimates of uncertainty (e.g. confidence intervals)
☐	☒	For null hypothesis testing, the test statistic (e.g. F, t, r) with confidence intervals, effect sizes, degrees of freedom and P value noted<br>*Give P values as exact values whenever suitable.*
☒	☐	For Bayesian analysis, information on the choice of priors and Markov chain Monte Carlo settings
☒	☐	For hierarchical and complex designs, identification of the appropriate level for tests and full reporting of outcomes
☒	☐	Estimates of effect sizes (e.g. Cohen's d, Pearson's r), indicating how they were calculated

Our web collection on statistics for biologists contains articles on many of the points above.

Software and code

Policy information about availability of computer code

Data collection	Data retrieved from the Sequence Read Archive (GenBank SRA)
Data analysis	Blast+ tools v2.6.0<br>HMMER v3.1b<br>Bowtie2 V 2.3 - read mapping<br>Geneious R8 - read mapping, visualisation, annotation<br>BedTools V2.27 - used for calculating coverage along each contig<br>HH-suite v3 - used for HMM searches<br>GeneMarkS-v2.5 - gene prediction<br>CLC genomics workbench v6 - genomic assembly<br>CLC assembly cell v4<br>MUSCLE v3.6 - multiple sequence alignment<br>PhyML V2.2.3<br>Glimmer 3 - gene prediction on complete virus genomes

For manuscripts utilizing custom algorithms or software that are central to the research but not yet described in published literature, software must be made available to editors and reviewers. We strongly encourage code deposition in a community repository (e.g. GitHub). See the Nature Research guidelines for submitting code & software for further information.

Data

Policy information about availability of data

All manuscripts must include a data availability statement. This statement should provide the following information, where applicable:

- Accession codes, unique identifiers, or web links for publicly available datasets
- A list of figures that have associated raw data
- A description of any restrictions on data availability

Data availability
All raw and processed genomic data, including original and reconstructed virus genomes from each location (fasta and annotated GFF files) and all gene variants from each MGI are available on figshare: doi.org/10.6084/m9.figshare.11881773. The metagenomic island gapclosing protocol is available on protocols.io: dx.doi.org/10.17504/protocols.io.bix8kfrw. Raw metagenomic reads are available from the GenBank Sequence Read Archive (www.ncbi.nlm.nih.gov/sra) using the accession numbers in Table 1. Virus metagenomic assembled genomes used is this study are available from GenBank under the Bioproject PRJNA283341 (www.ncbi.nlm.nih.gov/bioproject). Cryophage and the Mu-like cryoconite phage have been deposited in GenBank under the accession numbers MT820023 and MT820024.

Field-specific reporting

Please select the one below that is the best fit for your research. If you are not sure, read the appropriate sections before making your selection.

☒ Life sciences ☐ Behavioural & social sciences ☐ Ecological, evolutionary & environmental sciences

For a reference copy of the document with all sections, see nature.com/documents/nr-reporting-summary-flat.pdf

Life sciences study design

All studies must disclose on these points even when the disclosure is negative.

Sample size	No sample size calculation was performed as we used a dataset of previously assembled virus genomes from Bellas et al 2015 PMID: 26191051. These genomes represented known virus diversity found in cryoconite habitats and were assembed from the same metagenomes we used in this study.
Data exclusions	Virus genomes that recruited reads across <70% of their read length were excluded from metagenomic island detection. We established this criteria after analysis and manual inspection of mapped contigs in Geneious to ensure we could detect metagenomic islands with BedTools. Genomes which mapped reads to less than 70% of their length most often had insufficient coverage to successfully detect MGI regions. .
Replication	The genome reconsecrations can be reproduced by repeating the procedure described on the dataset, or independently by using a gapcloser module e.g SOAP de novo gapcloser, to close some of the metagenomic island gaps.
Randomization	Not relevant, conclusions are based on an analysis of all known genomes reconstructed from the same environment. We are not testing the outcome between treatments.
Blinding	Not relevant, conclusions are based on an analysis of all known genomes reconstructed from the same environment. We are not testing the outcome between treatments.

Reporting for specific materials, systems and methods

We require information from authors about some types of materials, experimental systems and methods used in many studies. Here, indicate whether each material, system or method listed is relevant to your study. If you are not sure if a list item applies to your research, read the appropriate section before selecting a response.

Materials & experimental systems

n/a	Involved in the study	
☒	☐	Antibodies
☒	☐	Eukaryotic cell lines
☒	☐	Palaeontology and archaeology
☒	☐	Animals and other organisms
☒	☐	Human research participants
☒	☐	Clinical data
☒	☐	Dual use research of concern

Methods

n/a	Involved in the study	
☒	☐	ChIP-seq
☒	☐	Flow cytometry
☒	☐	MRI-based neuroimaging
